# Effects of cognitive task complexity and online planning on second language learners’ argumentative writing

**DOI:** 10.3389/fpsyg.2023.1121994

**Published:** 2023-08-14

**Authors:** Ting Sophia Xu, Lawrence Jun Zhang

**Affiliations:** ^1^School of Literature and Media, Dongguan University of Technology, Dongguan, China; ^2^Faculty of Education and Social Work, University of Auckland, Auckland, New Zealand

**Keywords:** cognitive task complexity, online planning, EFL writing, argumentative writing, Limited Attentional Capacity Model, Cognition Hypothesis

## Abstract

Based on Kellogg’s writing model, Skehan’s Limited Attentional Capacity Model (LACM), and Robinson’s Cognition Hypothesis, our study investigated the effects of cognitive task complexity on syntactic complexity, lexical complexity, accuracy, fluency, and functional adequacy in Chinese L2 students’ argumentative writing, when students were under an online planning condition. Sixty-eight participants from a Chinese university were recruited to complete two writing tasks with task complexity varied in terms of [+ argument elements]. The findings showed that increasing task complexity led to decreased subordination in terms of clauses per T-unit and dependent clauses per clause, increased phrasal elaboration in terms of coordinate phrases per clause, and no changes in mean length of T-unit, T-units per sentence, mean length of clause, and complex nominals per clause. Neither significant differences in accuracy nor fluency were found as a function of increasing task complexity. Detrimental effects on functional adequacy in content, organization, and overall scores were identified with the increases in task complexity. The trade-offs between syntactic and lexical complexity and between syntactic complexity and functional adequacy support the basic principle of Skehan’s LACM that human’s information processing capacity is limited and Kellogg’s claim that learners have a limited central executive capacity in writing. Implications of the results of this research are discussed.

## Introduction

The cognitive processes of writing have been explored since the 1980s. Some scholars (e.g., Flower and Hayes, 1980; Bereiter and Scardamalia, 1987) proposed the writing models and identified the writing process in L1 writing, among which Kellogg’s (1996) writing model is well-known for the initial prediction of the cognitive demands placed by writing processes concerning working memory. Three processes of formulation, execution, and monitoring have been distinguished during L1 writing in Kellogg’s (1996) writing models, laying the foundation for understanding L2 writing processes. However, Kellogg’s model neither considers the influences of task characteristics on writing processes nor predicts how learners’ attentional resources are devoted to the writing processes as a function of varying task-generated cognitive demands.

In the context of the introduction of task-based language pedagogy, Skehan (1996a, 1998) and Robinson (2001, 2005, 2011a,b) respectively proposed two cognitive-interactionist models, the Limited Attentional Capacity Model and Cognition Hypothesis, to describe learners’ information processing and predict how task design factors influence task complexity, learners’ attentional allocation, and then learners’ complexity, accuracy, and fluency performance during oral task completion. Inspired by the two models of the Limited Attentional Capacity Model and Cognition Hypothesis and task-based research studies in speaking, an increasing number of empirical studies related to task complexity were sparked in L2 writing (Kuiken et al., 2005; Kuiken and Vedder, 2007a, 2008; Ong and Zhang, 2010; Johnson et al., 2012; Ong and Zhang, 2013; Cho, 2015; Rahimi, 2019; Rahimi and Zhang, 2019; Zhan et al., 2021; Xu T. et al., 2022; Xu Z. et al., 2022). However, the findings, with regard to the effects of task complexity in L2 writing, have been far from conclusive. The various operationalizations of task characteristics might be one of the potential reasons for the mixed findings, highlighting the need for replication studies with consistent task complexity manipulation (Johnson, 2017).

Another possible reason for the contradictory results is that the theoretical underpinnings of the Limited Attentional Capacity Model and Cognition Hypothesis, that were proposed for oral tasks, may not be accurately appropriate for writing scope (Johnson et al., 2012). Thus, there is a pressing need to understand learners’ information processing stages under the writing schemata by tying the cognitive task complexity models of Cognition Hypothesis and the Limited Attentional Capacity Model together with Kellogg’s writing models. Some scholars have pioneered such connections in L2 task-based research. For example, Kormos (2011) posited that task complexity can be inherent in the formulation stage of the writing process and place cognitive demands in some writing processes at a time or in an independent stage of the writing process depending on task complexity manipulations. More research is warranted to study task complexity with reference to the cognitive writing process, which helps understand how different task features and implementation conditions vary the amount of attention available and the focus of learners’ attentional resources when a message is expressed during composing.

Additionally, in the studies concerning the resource-dispersing features of task complexity, pre-task planning was the single most commonly investigated variable, with other planning types, such as online planning, scarcely being focused. Given pre-task planning and online planning impact different aspects of English as a foreign language (EFL) writing processes (Ellis and Yuan, 2004), online planning might play different roles when compared with pre-task planning in L2 writing when cognitive task complexity is changed. The involvement of different planning types, such as online planning, could add to our knowledge of where and how learners attend their attentional resources to different writing processes during task completion in L2 writing.

Informed by the gaps identified in the research literature, this study attempted to investigate the effects of cognitive task complexity on L2 learners’ argumentative writing in terms of syntactic complexity, lexical complexity, accuracy, fluency, and functional adequacy, when learners were under an online planning condition. Our study chose Kellogg’s (1996) writing model, Robinson’s (2001, 2005, 2011a,b) Cognition Hypothesis, and Skehan’s (1996a, 1998) Limited Attentional Capacity Model as the theoretical underpinnings to explain the complex nature of writing processes in the task-based L2 writing research. Our study is anticipated to contribute to the knowledge of how different task features and implementation conditions influence L2 learners’ cognitive resources allocation and then, their writing performance.

## Literature review

### Limited Attentional Capacity Model

The Limited Attentional Capacity Model was proposed based on the working memory theories (Gathercole and Baddeley, 1993; Carter, 1998), in its claim that learners “have a limited information processing capacity and must therefore prioritize where they allocate their attention” (Skehan, 2013, p. 189). When learners complete a cognitively demanding task and reach their attentional limits, trade-off effects will occur among the three dimensions of complexity, accuracy, and fluency (Skehan, 1996a; Skehan and Foster, 1999, 2001). Skehan (1996a, 1998) has suggested three principles to analyze the cognitive complexity of tasks: code complexity, cognitive complexity, and communicative stress. Code complexity is related to the linguistic demands of a task. A complex task is likely to require more advanced structures or greater densities of advanced structures. Cognitive complexity, including processing and familiarity, is concerned with the content or meaning of task performance. Processing refers to “the amount of on-line computation that is required while doing a task,” and is closely related to “the extent to which the learner has to actively think through task content” (Skehan, 1996a, p. 52). Familiarity refers to the extent to which participants possess, and can readily use task-related schematic information or knowledge to complete the task at hand. The greater the familiarity, the lower the task complexity, whereas the greater the processing demand, the higher the task complexity. Communicative stress is about the performance conditions, resulting from differences in time pressure, modality, scale, and participant variables.

These three dimensions determine task complexity, consequently influencing learners’ attentional allocation during task completion. For example, learners may become less willing to take risk of elaborating the interlanguage system and reduce their writing fluency when prioritizing accuracy in language performance. Learners’ focus on complexity may increase “the chances that new forms will be incorporated into interlanguage systems, promote risk-taking, and require attention being devoted to the new forms of language” with low priority being attached to fluency and accuracy (Skehan, 1996a, p. 50). Learners’ priority of fluency, such as emphasizing language accessibility, would be at the cost of the development of interlanguage system and the control of interlanguage system. Moreover, Skehan (2009) speculated that there might be competition for limited working memory between a high level of cognitive processing and the local linguistic form. For a task that demands more working memory to convey the content, learners’ attentional resources will be less available for language forms, leading to poor language performance in complexity and accuracy, which is especially true for EFL learners.

### Cognition Hypothesis

Robinson’s (2001, 2005, 2011a,b) Cognition Hypothesis states that learners’ attentional resources could be attached to language form to process the input more deeply and elaborately with increased cognitive demands of tasks, as learners have multiple attentional resource pools. Robinson (2001, 2005, 2011a,b) claimed that cognitive complexity could be operationalized across the three triads of task complexity, task conditions, and task difficulty that interact to influence learners’ learning and performance. Task complexity refers to the characteristics that elicit cognitive demands on learners. Two types of cognitive task features, resource-directing and -dispersing variables, comprise task complexity. The resource-directing variables, like few or many elements, [+ Here-and-Now], past or present events, and fewer or more reasoning demands, place cognitive and conceptual demands on participants, directing learners’ attention to particular aspects of the language code system and facilitating “the development and acquisition of new L2 form-concept mappings” (Robinson, 2007, p. 18). Resource-dispersing variables, like prior knowledge, planning time, and the number of tasks to complete, produce performative and procedural demands on learners’ cognitive resources, promoting learners’ fast real-time access to “an already established interlanguage system” (Robinson, 2007, p. 18). Increasing task complexity with resource-directing variables “has the potential to direct learners’ attentional and memory resources to the way the L2 structures and codes concepts” (Robinson, 2011a, p. 15), thus negatively affecting fluency, but positively influencing accuracy and complexity. Increasing task complexity with the manipulation of resource-dispersing variables can promote learners’ consolidation and automatic access to their existing interlanguage resources rather than the development and control of their interlanguage system.

Task condition includes participation and participant variables. Task difficulty refers to learners’ perceptions of task-generated cognitive demands. Across task complexity, task conditions, and task difficulty, task complexity should be the sole basis for sequencing decisions, whereas task difficulty and task conditions can be used to inform the online decisions on how to implement tasks (Robinson, 2001, 2005, 2011a,b).

### Kellogg’s writing model

**Figure 1:**
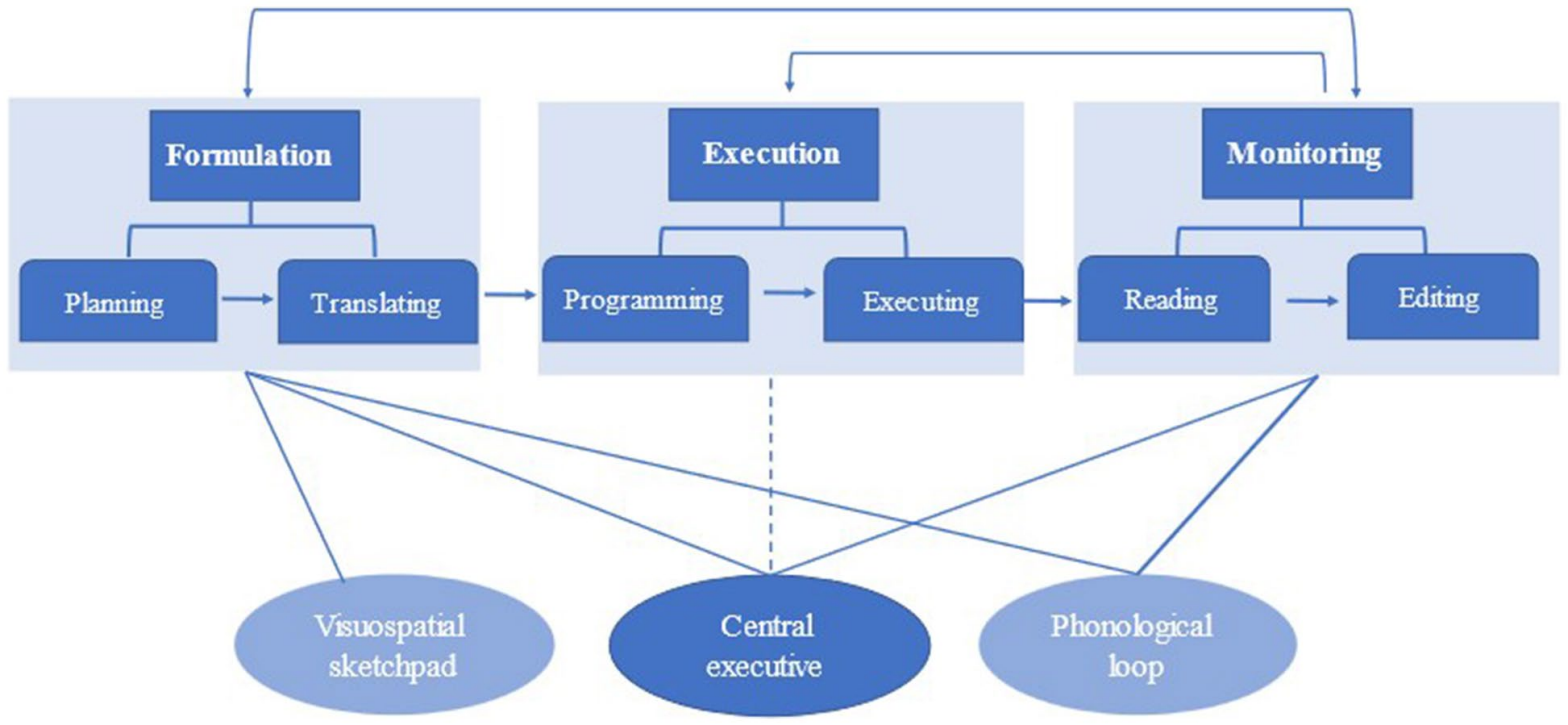
Kellogg’s (1996) model of writing process, from Kellogg (2013). Reproduced by permission of Taylor and Frances Group, LLC, a division of Informa plc.

In Kellogg’s (1996) writing model (see Figure 1), three different components have been distinguished in the writing process, with each comprising two sub-processes: formulation (planning and translating), execution (programming and executing), and monitoring (reading and editing).

The formulation system plans the content and translates ideas into sentences; during planning, writers use their working memory to establish goals and retrieve ideas and knowledge to write. Translation is “the amalgam of linguistic processes needed to convert an idea into a written message” (Kellogg, 1996, p. 60); the lexical, syntactic, and rhetorical items are selected to encode ideas into words during translating. In the execution system, while programming refers to adopting the appropriate motor system (e.g., typing, handwriting, or dictating) to translate the output, executing occurs when the production is created into words and sentences based on the translating process. Monitoring refers to reading and editing the produced version to ensure the writer’s intention is adequately expressed. During the editing, writers can either edit the localized errors, like spelling and diction, or the global problems, such as the paragraph and text organization issues.

Kellogg (1996) highlighted the significance of working memory capacity in writing. The central executive, a critical working memory component, is involved in all the sub-processes except executing. According to Kellogg (1996), learners have a limited capacity of central execution and have to compromise among three writing processes when under the pressure of completing cognitively demanding tasks, which is consistent with Skehan’s Limited Attentional Capacity Model.

### Empirical studies of task complexity

Robinson’s Cognition Hypothesis and Skehan’s Limited Attentional Capacity Model, although originally proposed for L2 speaking, have been used by a large number of empirical studies to examine the impacts of task complexity in L2 writing. Availability of content support (Kormos, 2011; Révész et al., 2017; Abrams, 2019), the number of elements to be considered (Kuiken et al., 2005; Kuiken and Vedder, 2007a, 2008; Cho, 2015; Rahimi, 2019; Rahimi and Zhang, 2019; Zhang and Zhang, 2021; Xu et al., 2023), and [+ Here-and-Now] (Ishikawa, 2007) are the resource-directing variables of task complexity that researchers have given a lot of attention to. In what follows, the studies, related to our research design, on the task complexity manipulated by changing the number of elements were reviewed.

Kuiken and his colleagues (Kuiken et al., 2005; Kuiken and Vedder, 2007b, 2008, 2012) investigated the effects of task complexity on L2 narrative letter writing in a set of studies (Kuiken et al., 2005; Kuiken and Vedder, 2007b, 2008, 2012). The complex tasks were to make a choice of a Bed and Breakfast from five options based on six criteria (six elements), while the simple tasks were about choosing a holiday resort according to three requirements (three elements). Participants’ syntactic complexity, lexical variation, and accuracy were assessed. The four studies found that increases in task complexity positively affected learners’ accuracy performance, but no significant effects on syntactic complexity were identified. Mixed findings of lexical performance were found in these studies.

To extend Kuiken and Vedder’s (2007b, 2008, 2012) studies, Frear and Bitchener (2015) made low, medium, and high task complexity. The low complex task asked participants, based on their own resources, to introduce New Zealand to a friend in the letter, which was expected to have a limited cognitive demand. The medium and complex task required participants to choose a restaurant for visiting friend(s) based on different amount of information about each restaurant and the friend’s requirements. Enhanced lexical complexity, but reduced ratio of adverbial dependent clauses to T-units were found as a function of increasing task complexity, indicating that participants may prioritize their limited cognitive resources to “the easier lexical means of meeting the pragmatic requirements of the tasks” over “the generation of grammar” (Frear and Bitchener, 2015, p. 53).

Later, Cho (2015) manipulated task complexity regarding more or fewer argument elements and varying reasoning demands within the topic of “choosing the best roommates” in a Korean EFL context. Participants’ argumentative writing was measured by syntactic complexity, accuracy, and fluency in both global and task-specific indices. The results showed that increased task complexity did not influence accuracy and syntactic complexity, but positively affected fluency. The findings contradict the prediction of Cognition Hypothesis, but support Skehan’s Limited Attentional Capacity Model. Cho (2015) argued that participants’ prioritization of fluency, according to Skehan (1996a), may interfere with attention to the dimensions of accuracy and complexity.

Likewise, Rahimi (2019) studied how cognitive task complexity impacted argumentative writing in an Iran EFL learning context, but with a different topic of project funding allocation. In addition to syntactic complexity, lexical complexity, accuracy, and fluency, text quality in terms of content, organization and overall quality was also measured. The results demonstrated that the increased complexity of the task led to enhancements in syntactic complexity, lexical complexity, content, organization, and quality of writing, which partially supports the predictions of Cognition Hypothesis. A significant negative impact on accuracy was, however, found. The trade-off between complexity and accuracy is aligned with Skehan’s Limited Attentional Capacity Model.

The relationship between planning and task complexity in L2 writing has been discussed in many studies (Ong and Zhang, 2010, 2013; Johnson et al., 2012). Planning, according to when it takes place, can be operationalized as pre-task planning and online planning (Ellis, 2005). Different planning types play various roles in learners’ writing process. For example, rehearsal, a type of pre-task planning, may assist writers in attending in both formulation and monitoring, leading to all-around improvements. Strategic planning, also a type of pre-task planning, may help learners mainly focus on conceptualization, which promotes better message complexity and increased fluency. Unpressured online planning may enable learners to devote attention to formulation and monitoring, contributing to a more accurate performance (Ellis, 2005). In the existing studies of task complexity, pre-task planning, rehearsal or strategic, has been increasingly studied, while there is a lack of research on the role of online planning in task complexity in L2 writing.

Ong and Zhang (2010, 2013) conducted a set of two studies to explore the effects of task complexity on L2 learners’ argumentative writing production. Task complexity was manipulated by three independent variables: varying planning conditions (20 min extended pre-task planning, 10 min pre-task planning, 30 min free writing, and a control group), + ideas and/or macrostructure support (topic, ideas and macro-structure available, topic and ideas available, and topic available), and + draft given during revision.^1^ Based on Robinson’s theory, Ong and Zhang supposed that task complexity decreased from free writing, to pre-task planning and then to extended pre-task planning conditions with planning time before task increased from 0 to 20 min. Participants’ lexical complexity, fluency, and writing quality were analyzed. The results showed increased task complexity contributed to significantly greater fluency, lexical complexity, and writing quality. In other words, the findings suggested that the provision of pre-task planning time might inhibit writers’ writing performance, contrary to previous studies in the field of L1 writing (Kellogg, 1987, 1990) or prior findings of EFL oral production (Skehan and Foster, 1997).

Of note, free writing cannot be defined as online planning, although participants were required to start writing immediately and write continuously for 30 min (Ong and Zhang, 2010, 2013). The concept of free writing in Ong and Zhang’s (2010, 2013) studies was adopted from Elbow’s (1973, 1981) and Galbraith and Torrance’s (2004) research. During free writing, learners were encouraged to do no planning before writing and write immediately and continuously what comes to their minds without considering how well the text is expressed or organized. In other words, continuous writing rather than online planning is what free writing emphasized, which was confirmed by Ong and Zhang’s (2010, 2013) conclusion that writers in the extended pre-task and pre-task planning conditions were more engaged in online planning than those in the free-writing condition.

Motivated by the contradictory findings, Johnson et al. (2012) investigated the effect of different sub-processes of pre-task planning on argumentative writing in terms of grammatical complexity, lexical complexity, and fluency. Five groups, with each group under a sub-pre-planning condition, were set: Idea generation planning group asked learners to list ideas on the topic; organization planning group to make an outline of the main ideas and the supporting information; goal setting planning group to list rhetorical goals; goal setting and organization planning group to list goals and make an outline; and control group to complete word match to cover the planning time. Neither lexical complexity nor grammatical complexity was found due to the provision of pre-task planning. Some inconsequential effects on fluency were identified with organization planners making shorter sentences than their counterparts in the control group. These findings are in contrast not only to the negative findings that pre-task planning reduced L2 writing fluency, lexical complexity, and writing quality (Ong and Zhang, 2010, 2013), but also Skehan’s Limited Attentional Capacity Model and Robinson’s Cognition Hypothesis. Johnson et al. (2012) speculated that Limited Attentional Capacity Model and Cognition Hypothesis, which were proposed for oral tasks, may not be accurately appropriate for writing scope, and the distinction between writing and speaking may make the inconsistent findings in the studies on pre-task planning in the L2 writing field.

Later, Rahimi and Zhang (2018) explored how cognitive task complexity and pre-task planning together affect EFL writing. The pre-task planning group (provision of 10-min pre-task planning) and control group (absence of 10-min pre-task planning) completed two tasks with writing task complexity manipulated in terms of [+ element]. The results showed that pre-task planning had no effects on accuracy or lexical complexity, but positive effects on fluency, syntactic complexity, content, and quality of writing regardless of task complexity. Increases in task complexity positively affected syntactic complexity, lexical complexity, content, and quality of writing, but negatively influenced accuracy and fluency regardless of planning conditions. Rahimi and Zhang (2018) concluded that simultaneously increasing task complexity and providing pre-task planning improved participants’ syntactic complexity, content, organization, and quality of writing, which supports Robinson’s Cognition Hypothesis, but is inconsistent with the previous findings (Ong and Zhang, 2010, 2013; Johnson et al., 2012).

Ellis and Yuan’s (2004) study was one of the very few that investigated online planning in L2 writing, but not under the umbrella of cognitive task complexity. The effects of pre-task planning, unpressured online planning, and no planning on EFL narrative written performance were compared in their study. The results showed that pre-task planning led to greater fluency, while unpressured online planning resulted in better accuracy. In contrast, the no-planning condition negatively influenced fluency, complexity, and accuracy. The results suggest that different planning types affected participants’ writing processes differently. Pre-task planning facilitated formulation process, while unpressured online planning assisted monitoring process. In contrast, writers with no-planning time had to deal with formulation, execution, and monitoring under pressure. The results, however, did not control the total time in each group, with pre-task planning group spending 27 min, online planning 21 min on average, and no-planning writers 17 min, which may act as confounding variables and consequently affect learners’ task performance.

To sum up, with L2 writing being the focus of research, neither the results of studies on task complexity manipulated by [+ few elements] (Kuiken et al., 2005; Kuiken and Vedder, 2007a, 2008; Cho, 2015; Rahimi, 2019; Rahimi and Zhang, 2019; Xu et al., 2023), nor on pre-task planning (Ong and Zhang, 2010, 2013; Johnson et al., 2012) have achieved consistency. The mixed findings indicated that the issue of how L2 learners allocate their attention when facing different levels of task complexity or under a planning condition during writing still remains to be resolved. The knowledge of the role of online planning, different from pre-task planning, in task complexity and L2 writing is very limited. These gaps support the need to explore how cognitive task complexity, together with online planning, affects L2 learners’ cognitive resources allocation and then writing performance.

The present study was guided by the research question as follows: What are the effects of cognitive task complexity on syntactic complexity, lexical complexity, accuracy, fluency, and functional adequacy, when L2 learners are provided online planning?

## Methods

### Participants

The participants were 68 L2 English learners from a university in China, and all of them regarded Chinese as their first language. Their ages ranged from 19 to 21, with a mean age of 20. Sixty-one of the participants were female, and 7 were male. The participants had an average of 11.23 years of English learning, and none had overseas learning experiences. All the participants had English as their major, and all courses were taught in English in their undergraduate program. At the time of the study, they were in their second-year study and had almost finished the required courses designed to develop their English competence in reading, writing, listening, and speaking, which means they were reaching an intermediate level of English proficiency, according to the requirements of Ministry of Education.

### Task

The two argumentative writing tasks (see Supplementary Appendix), a simple one and a complex one, were based on Xu Z. et al. (2022), Xu et al. (2023) tasks, which adapted topics and prompts from Cho’s (2015) study. The choice of “cooking” in Cho’s (2015) task prompts was replaced by an alternative option of “playing football” to suit the Chinese context. The tasks were presented in Chinese to ensure all the participants could easily understand the instructions. Xu Z. et al. (2022), Xu et al. (2023) have independently validated the task complexity manipulations by using dual-task methodology, expert judgments, and post-task questionnaire and proven the efficacy of the tasks that the complex task was more cognitively demanding than the simple version. The topic of the tasks is to choose the best roommates. The complexity of the tasks was manipulated in terms of more or fewer argument elements and different reasoning demands. The simple task asked learners to decide on one pair of the best roommates from four options based on different information about hobbies, personalities, studying styles, and sleeping patterns. In the complex task version, participants were instructed to make decisions of the best roommates, choosing two pairs from six options based on varying information of hobbies, personalities, studying styles, sleeping patterns, favorite subjects, and individual sanitary habits. The complex task, with one more pair of the best roommates to decide and more information on each roommate candidate, cost more cognitive demands than the simple one (Cho, 2015; Xu Z. et al., 2022, Xu et al., 2023).

### Procedure

Before the study, a pilot study was conducted to decide the writing time, where the slowest participants spent 35 and 37 min completing the simple and complex tasks, respectively, when no time limits were set. Forty minutes of writing time was finally decided, as it, on the one hand, was enough for participants to complete the tasks in no rush. On the other hand, it could prevent students from feeling no challenge and being less willing to effectively extend any goal of complexity, accuracy, or fluency in the absence of time limits.

The two writing tasks were completed in a counterbalanced way to avoid potential practice and fatigue effects: half the participants took the simple task first, and half took the complex one first. There was a one-month interval between the two tasks. The timeline and counterbalance of the two tasks are presented in Table 1. During writing, participants were instructed to begin writing immediately and plan while they were writing. The researcher and a research assistant monitored and reminded the participants who did not start writing within 5 min. It was assumed that participants would have ample time for online planning while they were writing based on the pilot study.

**Table 1:** Counterbalance of the two writing tasks.

Participants	First time (week one)	Second time (week five)
Half (*n* = 35)	Task 1	Task 2
Half (*n* = 33)	Task 2	Task 1

The tag of “simple task” and “complex task” was labeled as Task 1 and Task 2.

Participants were given a free option to take part in a retrospective interview after the completion of the tasks, five of whom voluntarily participated in the interview that was conducted on the day after the second writing task. Interview questions concerned students’ planning and writing focus during the writing and planning process. Participants were interviewed individually in a comfortable place (an empty classroom). All the contents or questions were explained in Chinese. Students were informed that their responses were recorded and they had the rights to skip any questions and have the recorder switched off at any time.

### Data coding and analysis

#### Analysis of writing performance

##### Syntactic complexity

To tap distinct and complementary dimensions of complexity, syntactic complexity was measured in four sub-constructs: overall complexity, subordination, phrasal elaboration, and coordination (Norris and Ortega, 2009). The overall complexity was assessed by the mean length of T-unit (MLT) that has consistently been used as a length-based measure (Frear and Bitchener, 2015; Ruiz-Funes, 2015; Yang et al., 2015; Yoon, 2017). Learners develop their ability to use phrasal-level complexification later than hypotaxis, such as subordination (Halliday and Mattiesen, 1999). Subordination, measured by clauses per T-unit (C/T) and dependent clauses per clause (DC/C), was considered a useful complexification index at an intermediate level. Phrasal-level elaboration, as a pervasive means to measure participants’ syntactic complexity, is an index of an advanced level in the field of writing (Norris and Ortega, 2009; Biber et al., 2011; Housen et al., 2019). Mean length of clause (MLC) was used for phrase-level measures, coordinate phrases per clause (CP/C) for phrasal coordination, and complex nominals per clause (CN/C) for noun phrase complexity. Complexity via coordination, measured by T-unit per sentence (T/S), was included as participants in the present study were L2 writers, whose English proficiency was lower than native speakers (Norris and Ortega, 2009). These seven measurements were analyzed by using the EFL Syntactic Complexity Analyzer (Lu, 2010).

##### Lexical complexity

Lexical complexity was measured by lexical density, the ratio of lexical words, and lexical diversity, the range of vocabulary used. Lexical words are “nouns, adjectives, verbs (excluding modal verbs, auxiliary verbs, ‘be’ and ‘have’), and adverbs with an adjective base, including those that can function as both an adjective and adverb (e.g., ‘fast’) and those formed by attaching the –*ly* suffix to an adjectival root (e.g., ‘particularly’)” (Lu, 2012, p. 192). To take text length into account, WT/√2 W^2^ was used to measure lexical diversity (Ong and Zhang, 2010). The number of words, word types, and lexical words was counted automatically by the EFL Lexical Complexity Analyzer (Lu, 2012).

##### Accuracy

Three general accuracy measurements, the number of errors per T-unit (Err/T), the number of errors per 100 words (EP100), and the ratio of error-free clauses (EFC/C), were employed to examine learners’ overall ability in using the second language. Errors were coded and tailed based on Polio and Shea’s (2014) guidelines. An error was considered as any digression in lexical choice, syntax, and morphology but not in capitalization or punctuation (Ellis and Yuan, 2004).

*Fluency.* Fluency was determined by the average number of words produced per minute to capture learners’ ability to automatically access their linguistic resources.

##### Functional adequacy

Functional adequacy is about the “successful task fulfillment” (Kuiken and Vedder, 2017, p. 596). With a focus on whether the pragmatic goals have been achieved, functional adequacy is independent from the linguistic dimension, such as CAF (Pallotti, 2009; Kuiken and Vedder, 2017). Students’ functional adequacy in terms of content, organization, and overall score was scored based on a composition rating scheme adopted by Jacobs et al.’s (1981) and Kuiken and Vedder’s (2017) criteria. More information about the rating criteria can be found in the Xu et al.’s (2023) study.

### Reliability of data coding and rating

The first author rated all the collected data for functional adequacy. A randomly selected 60% of the data were rated by a doctoral student majoring in applied linguistics, with the intraclass correlation coefficient for inter-rater reliability reaching 0.835 for the total score, 0.776 for the content, and 0.751 for the organization. As for the intra-rater reliability, the first author re-rated all the data 2 months apart, and the intraclass correlation coefficient was high, 0.881 for the total score, 0.837 for the content, and 0.790 for the organization.

With regard to errors, the first author coded all the data, and an over five-year experienced English writing lecturer coded 20% of the data separately. The identified errors marked by her and those identified by the first author were compared. The intraclass correlation coefficient reached 0.973.

### Statistical analysis

Paired-samples *t*-tests were used to compare the writing performance between the simple and complex tasks when the normality of the measures was met. The alpha level of *p* < 0.05 was set for all measures. Cohen’s *d* was reported as an indicator of the effect size (Cohen, 1992).

### Analysis of interview

Information on the students’ focus while writing, as well as planning, was gathered through the retrospective interviews. Based on participants’ responses and previous research (Manchón and De Larios, 2007), their focus during planning and writing was defined into three broad categories: Content, organization, and language. The categories cover the answers in which the words “content, organization, or language” were explicitly mentioned (e.g., my main focus is content) and those that implicitly indicated a certain focus (e.g., “I made the decision for the roommates first”). It is acknowledged that a single statement may contain more than one point of focus and that the three categories are sometimes interwoven in students’ statements. In such cases, the statement was coded according to the category represented in the statement. A single statement was, therefore, coded as more than one category. For example: “I will make an outline to figure out the logic, structure, and content.” In this case, the focus point was coded as “organization” and “content,” as the words “logic and structure” implicitly indicated the category “organization” and the word “content” was explicitly mentioned.

Students’ focus during online planning and writing were analyzed quantitatively and qualitatively. Quantitatively, the interviewee’s statements were coded, the number of the focus points provided in each statement was tallied, and the percentage of the three categories was calculated. The qualitative analysis mainly identified what students were doing during online planning and writing, thus providing explanations for the quantitative results.

## Results

### Effects on syntactic complexity

Table 2 displays the descriptive results for the seven syntactic complexity measures in the two writing tasks.

**Table 2:** Descriptive statistics for syntactic complexity.

Syntactic complexity	*N*	Simple task		Complex task	
		*M*	SD	*M*	SD
MLT	68	12.42	2.47	11.82	2.52
C/T	68	1.52	0.26	1.41	0.23
DC/C	68	0.33	0.10	0.28	0.10
T/S	68	1.10	0.09	1.11	0.10
MLC	68	8.17	0.88	8.38	1.14
CP/C	68	0.31	0.11	0.36	0.13
CN/C	68	0.85	0.19	0.84	0.23

MLT, mean length of T-unit; C/T, clauses per T-unit; DC/C, dependent clauses per clause; T/S, T-units per sentence; MLC, mean length of clause; CP/C, coordinate phrases per clause; CN/C, complex nominals per clause.

A series of paired-samples *t*-tests was conducted to investigate the effects of task complexity on syntactic complexity when participants were under the online planning condition. The results showed that no significant difference was found in MLT between the simple (*M* = 12.42, SD = 2.47) and complex tasks (*M* = 11.82, SD = 2.52), *t* (67) = 1.909, *p* = 0.061, *d* = 0.238; in T/S between the simple (*M* = 1.10, SD = 0.09) and the complex tasks (*M* = 1.11, SD = 0.10), *t* (67) = −0.528, *p* = 0.599, *d* = −0.08; in MLC with the simple task (*M* = 8.17, SD = 0.88) versus the complexity task (*M* = 8.38, *SD* = 1.14), *t* (67) = −1.535, *p* = 0.130, *d* = −0.206; or in CN/C between the simple (*M* = 0.85, SD = 0.19) and complex tasks (*M* = 0.84, SD = 0.23), *t* (67) = 0.427, *p* = 0.671, *d* = 0.054. The effect sizes ranged as small.

Online planners used significantly more C/T in the simple task (*M* = 1.52, SD = 0.26) than in the complex task (*M* = 1.41, SD = 0.23), *t* (67) = 3.252, *p* = 0.002, *d* = 0.458, with the effect size close to medium. Likewise, students’ DC/C performance for the simple task (*M* = 0.33, SD = 0.10) was significantly greater than for the complex task (*M* = 0.28, SD = 0.10), *t* (67) = 3.228, *p* = 0.002, *d* = 0.446, with the effect size close to medium. In contrast, online planners produced significantly more CP/C in the complex task (*M* = 0.36, SD = 0.13) than in the simple one (*M* = 0.31, SD = 0.11), *t* (67) = −2.919, *p* = 0.005, *d* = −0.427, and the effect size was close to medium. These findings suggest that increasing task complexity led to a significant reduction in participants’ C/T and DC/C, but a marked increase in CP/C, with no significant influence on MLT, T/S, MLC, and CN/C.

### Effects on lexical complexity

Table 3 displays the descriptive results for the online planners’ lexical complexity performance in the two writing tasks.

**Table 3:** Descriptive statistics for lexical complexity.

Lexical complexity	*N*	Simple task		Complex task	
		*M*	SD	*M*	SD
LD	68	0.509	0.03	0.512	0.03
WT/√2 W	68	5.56	0.52	5.40	0.53

LD, lexical density; WT, word type; W, the number of words.

Paired-samples *t*-tests revealed no significant differences in lexical density between the simple (*M* = 0.509, SD = 0.03) and complex tasks (*M* = 0.512, SD = 0.03), *t* (67) = −0.740, *p* = 0.462, *d* = −0.097, when participants were under the online planning condition. The effect size was small. However, online planners’ WT/√2 W production in the complex task (*M* = 5.40, SD = 0.53) was statistically lower than their performance in the simple task (*M* = 5.56, SD = 0.52), *t* (67) = 2.960, *p* = 0.004, *d* = 0.320, with a small effect size. These results suggest that students’ lexical diversity in terms of WT/√2 W was inhibited by increased task complexity.

### Effects on accuracy

The descriptive results for the three accuracy measures in the simple and complex tasks were presented in Table 4.

**Table 4:** Descriptive statistics for accuracy.

Syntactic complexity	*N*	Simple task		Complex task	
		*M*	SD	*M*	SD
Err/T	68	0.74	0.29	0.73	0.37
EP100	68	0.060	0.02	0.061	0.03
EFC/C	68	0.63	0.12	0.63	0.15

Err/T, the number of errors per T-unit; EP100, the number of errors per 100 words; EFC/C, ratio of error-free clauses.

No significant differences were detected in either Err/T between the simple (*M* = 0.74, SD = 0.29) and complex tasks (*M* = 0.73, SD = 0.37), *t* (67) = 0.449, *p* = 0.665, *d* = 0.060, in EP100 with the simple task (*M* = 0.060, SD = 0.02) versus the complex task (*M* = 0.061, SD = 0.03), *t* (67) = −0.318, *p* = 0.752, *d* = −0.038, or in EFC/C between the simple task (*M* = 0.63, SD = 0.12) and the complex task (*M* = 0.63, SD = 0.15), *t* (67) = 0.105, *p* = 0.917, *d* = 0.014. These findings indicated that increases in task complexity had negligible effects on online planners’ accuracy performance.

### Effects on fluency

A paired-samples *t*-test was applied to examine the effects of task complexity on fluency. The findings showed that students’ fluency was not markedly different between their simple (*M* = 5.88, SD = 0.92) and their complex task completion (*M* = 5.89, SD = 1.04), *t* (67) = −0.018, *p* = 0.985, *d* = −0.002. The effect size was small.

### Effects on functional adequacy

Table 5 presents the descriptive results for functional adequacy measures in the two writing tasks when participants were under the online planning condition.

**Table 5:** Descriptive statistics for functional adequacy.

Syntactic complexity	*N*	Simple task		Complex task	
		*M*	SD	*M*	SD
Content	68	18.55	1.44	18.03	1.38
Organization	68	13.99	1.28	13.60	1.30
Overall score	68	64.38	4.05	63.14	4.48

Paired-samples *t*-tests revealed that the content of the online planners was better in the simple task (*M* = 18.55, SD = 1.44) than the complex one (*M* = 18.03, SD = 1.38), *t* (67) = 3.341, *p* = 0.001, *d* = 0.370, with a small effect size. The same pattern was found in organization with scores in the simple task (*M* = 13.99, SD = 1.28) higher than those in the complex task (*M* = 13.60, SD = 1.30), *t* (67) = 2.224, *p* = 0.030, *d* = 0.302; the effect size was small. Likely, online planners’ overall scores were outperformed in the simple task (*M* = 64.38, SD = 4.05) than the complex task (*M* = 63.14, SD = 4.48), *t* (67) = 2.459, *p = 0*.017, *d* = 0.290; effect size was small. These findings suggest that increasing task complexity had a detrimental effect on students’ functional adequacy in content, organization, and overall score.

### Interview

Four of the five interviewees reported they first spent a little time reading the instructions, quickly understanding the task requirements, and then started writing. Only one interviewee acknowledged that she made a general plan in her mind (maybe 5 min) after understanding the prompts and before beginning to write. All of them stated they had enough time to do online planning during writing, while none reported they had edited the essays after finishing writing. From the responses, it is apparent that the pre-task planning was limited, and online planning occurred during writing; this was the intention of the research design of this study.

With regard to the question of what they focused on during online planning, they mentioned content four times (30.77%), organization once (7.69%), and language eight times (61.54%). Online planners’ main focus was language, followed by content, and a small proportion of time spent on organization.

Two participants (#1 and #3) gave a very general explanation, such as “language expression is my main focus,” with very few about how they planned the language during online planning and writing. One participant (#4) stated that she carefully considered word choices and sentence structures during her online planning and writing. Another two (#2 and #5) reported they had a specific focus on language complexity, acknowledging that they tried to use more complex words and sentence patterns, as the following excerpts illustrate:

“I tried to search for some complex sentence patterns and sophisticated vocabulary, such as the words with more than three syllables, in my mind.”—#2

“During writing, I mainly focused on language expression. I tried to use some complex sentence constructions and native expressions.”—#5

It was interesting that neither the explicit words, “accuracy” and “grammar,” nor the implicit expressions of accuracy were detected in the interview data, although most attention was paid to the language category.

## Discussion

The results showed that increased task complexity led to a decrease in C/T and DC/C, and an increase in CP/C, although no significant influences on MLT, T/S, MLC, and CN/C. Ostensibly, the decline in subordination in terms of C/T and DC/C is contrary to Robinson’s (2001, 2005, 2007) Cognition Hypothesis, since increasing task complexity negatively affected the number of students’ subordinate clauses. However, it should be taken into account that learners develop their ability to use phrasal-level complexification later than subordination (Halliday and Mattiesen, 1999). The increases in phrasal-level elaboration (i.e., CP/C) but decreases in subordination (i.e., C/T and DC/C) indicate that online planners in the complex task produced more advanced language, with “lower levels of subordination” but “more complex phrases” (Norris and Ortega, 2009, pp. 562–563) than in the simple task.

The favorable results of syntactic complexity in the complex task, essentially, echo Robinson’s (2001, 2005, 2007) Cognition Hypothesis. As assumed by Robinson, learners’ attention and effort are expected to be channeled to the language code system to meet the increasing conceptual demands, consequently facilitating interlanguage development, when task complexity is augmented with more elements and more reasoning demands involved. It is plausible that students, with the help of online planning and increased task complexity, might be encouraged to use new structures, or to extend their interlanguage system, to the next level of syntactic development in L2 writing to express their meanings. Evidence can be found in the interview data that online planners focused more on language, such as considering word choice and sentence structures and selecting complex syntactic frames to encode their ideas.

The findings of syntactic complexity also corroborate the results for phrase-level complexity reported in the previous studies (Biber et al., 2011; Lu, 2011; Bulté and Housen, 2014; Mazgutova and Kormos, 2015; Yoon, 2017), proving the sensitivity and validity of the phrase-level elaboration as an indicator of syntactic development in EFL writing. The results of our study highlight the necessity to use syntactic constructs in different dimensions to capture a multi-dimensional and dynamic picture of L2 learners’ writing development (Norris and Ortega, 2009; Yang et al., 2015; Yoon, 2017).

Increasing task complexity reduced participants’ lexical complexity production in terms of WT/√2 W. This might be because the increased cognitive demands augmented participants’ pressure on working memory to complete the task. Cognitive task complexity is supposed to be inherent in the formulation stage of L2 writing process (Kormos, 2011), and therefore competition for working memory within the *translating* sub-process (i.e., between selecting lexical units and building syntactic frames) may occur when task complexity is increased. In view of the findings with regard to syntactic complexity and lexical complexity, it seemed that fewer attentional resources were available for online planners to retrieve advanced lexical units with more working memory attended to the syntactic structures, when they completed the complex task. As a result, they were more likely to employ familiar or simple words to express messages in the complex task, compared to the simple task, resulting in a decline in lexical diversity but an increase in syntactic complexity, which was evidenced in participants’ essays. For example, in a participant’s essays, the word “personalities” has been repeatedly used in the complex task, while synonyms, like “traits,” “personalities,” and “characteristics” have been employed to convey meanings in the simple task. Our findings also extend Frear and Bitchener’s (2015) hypothesis that there may be a trade-off between syntactic and lexical means of expression when learners experience pressure which is “brought to bear on limited attentional resources by cognitive task complexity” and pressure “of producing language that was not fully automatized” (p. 53).

No significant changes in any measures of accuracy were found between the simple and complex tasks. The results indicate that online planners devoted similar attentional resources to monitor linguistic accuracy when completing the simple and complex task versions. No emphases on accuracy in the interview might provide some evidence for the unnoticed differences in the production of accuracy between the simple and complex tasks. This finding is contrary to Skehan’s LACM, as no trade-off between accuracy and complexity (either syntactic or lexical complexity) was identified.

Increasing task complexity resulted in a significant decrease in functional adequacy performance in terms of content, organization, and overall score. There appears to be a trade-off between syntactic complexity and functional adequacy, which lends support to the LACM, that learners’ attentional resources are limited in capacity, although LACM does not make explicit predictions regarding the effect of task complexity on functional adequacy. Some scholars have suggested trade-off effects between learners’ linguistic outcomes and higher-order dimensions of EFL production. For instance, Kuiken and Vedder (2008) posited that learners’ favorable linguistic outcomes might be achieved with low priority being attended to the higher-order language processes. Pallotti (2009) speculated that learners might produce structures syntactically complex or accurate at the cost of lacking pragmatical adequacy. Similarly, Kormos (2011) also expressed a concern about the potential competition for the attentional resources of L2 writers, which may occur between syntactic encoding and text global organization during the writing process and between linguistic accuracy and discourse structure during monitoring process.

Kellogg’s (1996) writing model seems to provide some insights into such trade-off effects. The trade-off between learners’ linguistic output and higher-order performance may result from the pressure on working memory between the sub-processes of formulation (i.e., between *planning* and *translating*) that was triggered by some task implementation conditions, such as the increased task complexity and/or the provision of online planning. In our study, online planning seemed to help learners put the most attention (61.54%) to linguistic production during writing, with less attention devoted to content (30.77%) and organization (7.69%), as reflected in the responses in the interview. Increased task complexity may have greatly augmented online planners’ pressure on working memory which has a limited capacity (Skehan, 1996b). When the competition between *planning* and *translating* became increasingly obvious with the increases in task complexity, online planners prioritized *translating* with inability to engage in planning the content and organization. In other words, online planners in our study were probably pressured to focus more on form at the expense of achieving semantic and pragmatic goals when the writing task was cognitively taxing.

A significant correlation was found between WT/√2 W and the overall score in the present study (*ρ* = 0.346, *p < 0.001*), which corroborates Lu’s (2012) findings, when analyzing large-scale data from a corpus of Chinese learners, that Chinese students’ lexical diversity is significantly correlated to the raters’ judgment of the task quality. In light of this, the decline in the overall quality of the complex task might be related to the decreasing lexical diversity.

Increases in task complexity did not yield any significant differences in fluency. As previously discussed, online planners are likely to prioritize restructuring, which gets attention to the new forms of language, with accuracy and fluency being secondary, and adequacy possibly the last. This may explain why increasing the complexity of a task has not led to any significant changes in learners’ accuracy or fluency, but a significant decline in functional adequacy.

To conclude, the favorable findings in syntactic complexity, with increases in phrasal-level elaboration (i.e., CP/C) and decreases in subordination, resulted from the increased task complexity support Robinson’s Cognition Hypothesis. However, given that writing is a cognitive activity with recursive processes (Kellogg, 1996; Weigle, 2002), the relationship between task complexity and writing should be considered with a dynamic and comprehensive view rather than a segmentary way. When we take the results of syntactic complexity, lexical complexity, accuracy, fluency, and functional adequacy as a whole (see Table 6), trade-offs between syntactic and lexical complexity and between syntactic complexity and functional adequacy were identified with the increased task complexity. The overall result pattern lends support to Skehan’s (2013) LACM that “humans have a limited information processing capacity and must therefore prioritize where they allocate their attention” (p. 189). The results indicate that tensions may exist between syntactic and lexical complexity, and between form and pragmatic goals. It can be inferred that the tension in the formulation stage might be raised as a function of increasing task complexity, which probably leads to the competition for working memory between planning and translating, or within the translating process (i.e., between selecting lexical units and building syntactic frames). This interpretation, however, should be treated with caution, as think-aloud was not used to record the real-time data during online planning and writing.

**Table 6:** Result patterns for the effects of increasing task complexity on EFL writing performance.

Syntactic	Accuracy	Lexical	Adequacy	Fluency
+	=	−	−	=

+ means that increased task complexity had a positive effect, = means a non-significant effect, − means a negative effect.

## Conclusion

This study aimed to explore the effects of cognitive task complexity on Chinese L2 students’ argumentative writing in terms of syntactic complexity, lexical complexity, accuracy, fluency, and functional adequacy, when students were asked to start writing immediately and do online planning while writing. Increasing task complexity led to a trade-off between syntactic and lexical complexity, and between syntactic complexity and adequacy. No trade-off effect between complexity (either syntactic complexity or lexical complexity) and accuracy was found. The results indicate that tensions may exist between syntactic and lexical complexity, and between the local linguistic output (i.e., syntactic complexity) and the higher-order process (i.e., functional adequacy) when L2 students were completing the complex task under the online planning condition.

Theoretically, the overall trade-off result pattern lends support to the basic principle of Skehan’s (2013) LACM that “humans have a limited information processing capacity and must therefore prioritize where they allocate their attention” (p. 189). Furthermore, this study contributes to the enrichment of empirical evidence and theory on cognitive task complexity by introducing functional adequacy, independent from CAF, into the research, which leads to an understanding of the relationship between higher-order process and local linguistic output during the argumentative writing process.

When task complexity was increased for online planners, tensions between planning and translating sub-processes, and between lexical and syntactic encoding seemed to be triggered, resulting in the trade-offs between syntactic complexity and functional adequacy, and between lexical and syntactic complexity. These findings echo Kellogg’s (1996) writing model that learners’ central executive capacity is limited and also advanced our knowledge of how learners under the online planning condition compromised their working memory among the writing processes and sub-writing processes when under the pressure of completing the cognitively demanding argumentative tasks. Informed by Kormos (2011) speculation that task complexity is inherent in the formulation stage of writing, this study connected Kellogg’s writing model with the cognitive-interactionist models, Robinson’s Cognition Hypothesis and Skehan’s LACM, to explore the complex nature of writing processes in the task-based L2 writing. The results provided empirical insights into understanding and analyzing cognitive task complexity in L2 argumentative writing with reference to the writing processes of formulation, execution, and monitoring.

Methodologically, our study highlighted the necessity of using multi-dimensional linguistic measures and functional adequacy to describe the task and proficiency-related variation. For example, indices measuring syntactic complexity from overall complexity, subordination, phrasal elaboration, and coordination proved to be very useful in capturing the effects of task complexity on syntactic complexity. If phrasal elaboration was not tested in the present study, the results of syntactic complexity would be interpreted in a contradictory way with the decline in subordination in terms of C/T and DC/C. Likewise, the involvement of functional adequacy helps researchers capture the trade-off between the form (i.e., syntactic complexity) and pragmatic goals. Also, the multi-dimensional writing measures could allow teachers to comprehensively evaluate L2 learners’ writing.

Pedagogically, this study provided some guidance on making task-grading and sequencing decisions for EFL teachers when they schedule a syllabus for a given course considering the complexity of EFL argumentative writing (Zhang, 2021). Increasing task complexity could trigger pressure in the formulation stage of writing, and learners had the potential to devote more attention to language coding during online planning, which may assist the translating process primarily. Teachers may tailor task complexity to meet the learners’ needs analysis, encouraging students to use new structures and extend the interlanguage system with the help of providing online planning strategies. The tension between the local linguistic output and the higher-order process found in the argumentative writing in our study should also attract teachers’ attention. Teachers may design and sequence the argumentative tasks based on learners’ proficiency to minimize the negative effects of the increased cognitive demands, promoting a balanced writing development in complexity, accuracy, fluency, and functional adequacy (Skehan, 1998; Robinson, 2010).

Limitations need to be mentioned for studies in the related field to consider in the future. First, participants recruited in our study all regarded Chinese as their first language and were from a single Chinese university, which limit the generalizability of the findings. Also, to ensure that all participants were, to a large extent, under online planning during writing, only 68 L2 learners were included. Further studies are encouraged to include a larger sample size of participants from diverse L2 learning contexts, so that an understanding of the effects of cognitive task complexity and online planning on L2 learners’ argumentative writing can be generalized and broadened.

Second, the research attempted to find out the effects of task complexity on L2 writing when participants were provided online planning. The result pattern under the online planning condition was found, but no control group was included in the study, making it impossible to compare students’ performance under different planning conditions as a function of increasing task complexity. Further studies could involve different planning types, like pre-task planning and a control group. In this way, interactions between task complexity and planning conditions and the impacts of different planning types on writing processes could be explored, which helps advance the knowledge of how learners allocate their cognitive resources during the writing process.

Third, only the argumentative genre is included in the current study. In this way, findings may not apply to other types of writing beyond argumentative writing. The inclusion of various writing genres is warranted to explore the applicability and generalization of our findings to other types of writing.

Finally, qualitative data of what participants were doing and focusing on during online planning and writing were collected after the second task at one point in time. The retrospective interview cannot track what learners are actually doing during writing and online planning, as writing and planning are dynamic processes. Further research using the think-aloud approach is recommended to track all the real-time data (Rose et al., 2019; Zhang and Zhang, 2020), which helps deepen the understanding of how task complexity and online planning influence learners’ attentional allocation during task completion.

The effects of cognitive task complexity on L2 writing need further exploration, with mixed findings identified in the existing studies. This research could be replicated in future research, as there are a limited number of studies either using multi-dimensional writing measurements or focusing on the relationship between task complexity and online planning in L2 writing. This study could also be extended by further cognitive task complexity related research, involving other variables, such as different proficiency levels, task types, and task modalities.

## Data availability statement

The original contributions presented in the study are included in the article/Supplementary material, further inquiries can be directed to the corresponding author.

## Ethics statement

The studies involving human participants were reviewed and approved by The University of Auckland Human Ethics Committee. The patients/participants provided their written informed consent to participate in this study.

## Author contributions

TX and LZ contributed equally to the study in terms of research design, data collection, analysis, interpreting and writing it up, with LZ finalizing the manuscript for submission as the corresponding author. All authors contributed to the article and approved the submitted version.

## Funding

This work was supported by a joint scholarship for doctoral study offered to TX by The University of Auckland of New Zealand and the China Scholarship Council of the Ministry of Education of China.

## Conflict of interest

The authors declare that the research was conducted in the absence of any commercial or financial relationships that could be construed as a potential conflict of interest.

## Publisher’s note

All claims expressed in this article are solely those of the authors and do not necessarily represent those of their affiliated organizations, or those of the publisher, the editors and the reviewers. Any product that may be evaluated in this article, or claim that may be made by its manufacturer, is not guaranteed or endorsed by the publisher.

## Footnotes

^1^Revision conditions, unrelated to planning, are not reviewed in this part.

^2^WT = word type, W = the number of words.
