# Supplementary material for: Effects of cognitive task complexity and online planning on second language learners’ argumentative writing
Source: Front Psychol. 2023 Aug 14;14:1121994. doi: 10.3389/fpsyg.2023.1121994 (PMC10469471; doi:10.3389/fpsyg.2023.1121994)
Supplement: Supplementary file 1 [file Data_Sheet_1.docx]

**Appendix** Writing Tasks

***Task 1 (simple task***^[[1]](#footnote-1)^***)***

Imagine you can choose two best roommates from the four candidates when the new semester starts. Four properties of candidates: hobbies, personality, sleeping patterns, and studying style are listed in the table as follows.

Read the descriptions carefully and make a considered choice of the best roommates. Write an argumentative essay. Please provide at least 3 reasons to make your decision clear and convincing.

You have no time for pre-task planning but 40 minutes for writing and online planning. You should plan while you are writing. Please write at least 250 words.

The best roommates you choose: _________ & __________

| Name  Property | Nannan | Beibei | Niuniu | Dongdong |
| --- | --- | --- | --- | --- |
| Hobbies | Watching movies;  Listening to music;  Reading books | Listening to music;  Blogging;  Playing the guitar | Photography;  Playing football;  Traveling | Listening to music;  Playing football;  Playing computer games |
| Personality | Introvert;  Good at listening to others;  Not good at communicating | Outgoing;  Positive and enthusiastic;  Happy to help others;  Sometimes, ignoring others’ feelings unconsciously | Like playing with others;  Not good at listening to others | Outgoing;  Humorous;  Weak self-discipline |
| Sleeping pattern | Early to bed, early to rise | Late to bed, early to rise | Early to bed, late to rise | Late to bed, late to rise |
| Studying style | He/she likes to study in a quiet place on his/her personal. | He/she likes to read out when he/she is studying. | He/she likes to study with others. | He/she likes to study on his/her personal. |

***Task 2 (complex task)***

Imagine you can choose four best roommates from the six candidates when the new semester starts (two students in each dormitory). Six properties of candidates: hobbies, personality, sleeping patterns, studying style, favourite subjects and individual sanitary habits are listed in the table as follows.

Read the descriptions carefully and make a considered choice of the best roommates. Write an argumentative essay. Please provide at least 3 reasons to make your decision clear and convincing.

You have no time for pre-task planning but 40 minutes for writing and online planning. You should plan while you are writing. Please write at least 250 words.

The best roommates you choose: _________ & __________, ___________ & _________

| Name  Property | Nannan | Beibei | Niuniu | Dongdong | Kangkang | Xuxu |
| --- | --- | --- | --- | --- | --- | --- |
| Hobbies | Watching movies;  Listening to music;  Reading books | Listening to music;  Blogging;  Playing the guitar | Photography;  Playing football;  Traveling | Listening to music;  Playing football;  Playing computer games | Playing the piano;  Listen to music;  Watching movies | Playing football;  Playing computer games;  Playing magic |
| Personality | Introvert;  Good at listening to others;  Not good at communicating | Outgoing;  Positive and enthusiastic;  Happy to help others;  Sometimes, ignoring others’ feelings unconsciously | Like playing with others;  Not good at listening to others | Outgoing;  Humorous;  Weak self-discipline | Shy;  Like to play with others;  Weak self-discipline | Like to communicate with others and also good at listening to others;  Outgoing, but sometimes kind of noisy |
| Sleeping pattern | Early to bed, early to rise | Late to bed, early to rise | Early to bed, late to rise | Late to bed, late to rise | Early to bed, late to rise | Late to bed, early to rise |
| Studying style | He/she likes to study in a quiet place on his/her personal. | He/she likes to read out when he/she is studying. | He/she likes to study with others. | He/she likes to study on his/her personal. | He/she likes to study in a quiet place on his/her personal. | He/she likes to read out in a low voice, when he/she is studying. |
| Favourite subjects | English, Chinese | Math, Music | Sports, Arts | Computers, Music | Music English | Math, Computer |
| Individual sanitary habits | Tidy, but not good at clean | Good at clean | Neutral | Good at clean | Not good at clean | Neutral |

1. The words of “simple task” or “complex task” were not shown in students’ writing prompts. [↑](#footnote-ref-1)
